# Supplementary material for: Evidence-Based Severity Assessment of Animal Models for Pancreatic Cancer
Source: Biomedicines. 2024 Jul 5;12(7):1494. doi: 10.3390/biomedicines12071494 (PMC11275077; doi:10.3390/biomedicines12071494)
Supplement: Supplementary file 1 [file biomedicines-12-01494-s001.zip › 240516-Supplementals_PDA_final.pdf]

*Supplementary Materials to the article*

# Evidence-Based Severity Assessment of Animal Models for Pancreatic Cancer

**Tim Schreiber[1], Ingo Koopmann[1], Jakob Brandstetter[1], Steven R. Talbot[2], Lea Goldstein[1], Lisa Hoffmann[1], Anna Schildt[3], Markus Joksch[4], Bernd Krause[4], Robert Jaster[5], Rupert Palme[6], Dietmar Zechner[1], Brigitte Vollmar[1] and Simone Kumstel[1]*

[1] Rudolf-Zenker-Institute of Experimental Surgery, Rostock University Medical Center, Rostock, Germany
[2] Institute for Laboratory Animal Science, Preclinical Data Science, Hannover Medical School, Hannover, Germany
[3] Core Facility Multimodal Small Animal Imaging, Rostock University Medical Center, Rostock, Germany
[4] Department of Nuclear Medicine, Rostock University Medical Center, Rostock, Germany
[5] Division of Gastroenterology, Department of Medicine II, Rostock University Medical Center, Rostock, Germany
[6] Unit of Physiology, Pathophysiology and Experimental Endocrinology, Department of Biomedical Sciences, University of Veterinary Medicine, Vienna, Austria
* Correspondence: simone.kumstel@uni-rostock.de; Tel.: +49 381 494 2512

**Figure S1.** Data for tumor progression and burden.

**Figure S2.** Comparison of $RELSA_{max}$ values for sex related differences.

**Table S1.** Clinical score sheet.

**Table S2.** Severity assessment parameters for cancer cell injection in the orthotopic model divided by sex.

**Table S3.** Severity assessment parameters for cancer cell injection in the intravenous model divided by sex.

**Table S4.** Severity assessment parameters for cancer cell injection in the subcutaneous model divided by sex.

**Table S5.** ANOVA tables for analysis of severity assessment parameters on perioperative days (Figure 2).

**Table S6.** ANOVA tables for analysis of severity assessment parameters for tumor progression (Figure 3).

**Table S7.** ANOVA tables for analysis of severity assessment parameters for humane endpoint phase (Figure 4).

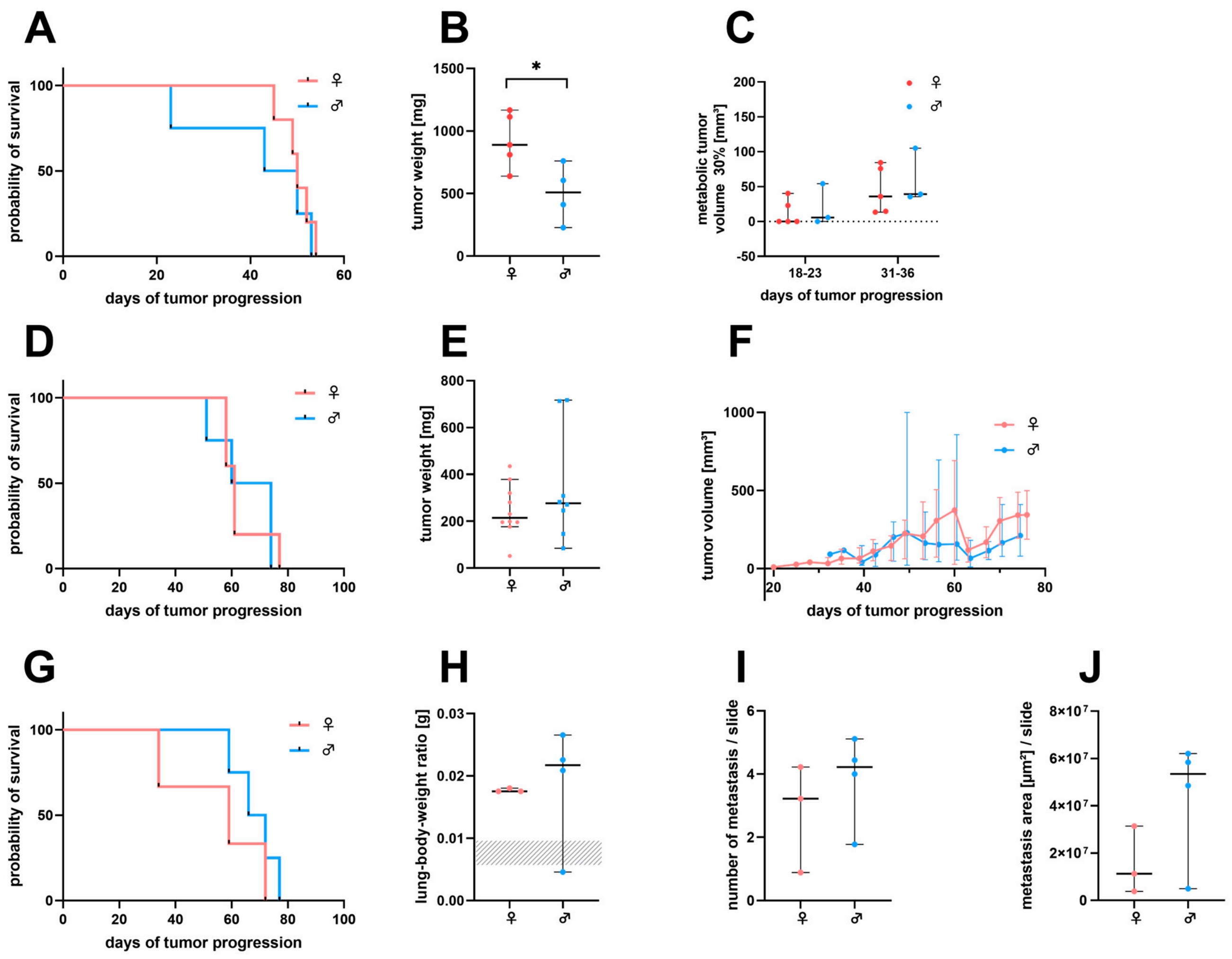


**Figure S1.** Data for tumor progression and burden were analyzed for sex related differences in PDA models induced by orthotopic (A-C), subcutaneous (D-F) and intravenous (G-J) cell injection. For all models probability of survival is shown (A, D, G). Tumor burden in the orthotopic and subcutaneous model is characterized either by tumor weight (B and E), tumor volume (F) or metabolic volume (30 %) quantified by the uptake of $^{18}$F-FDG via PET-CT (C). Metastatic burden of the intravenous model was evaluated with lung to body weight ratio (H) and metastasis count (I) and size (J) of H/E stained lung slices. Significant difference (*$p \leq 0.05$) was seen for orthotopic tumor weight on the day of euthanasia for male and female by unpaired t-test (B: df=7, t=2.811, p=0.0261). For male and female comparisons, no significant differences were observed for survival in all models by log-rank-test (A: $X^2$=0.4952, df=1, p=0.4816; D: $X^2$=0.0200, df=1, p=0.8875; G: $X^2$=0.9665, df=1, p=0.3256), for metabolic tumor volume 30 % in the orthotopic model by two-way ANOVA (C), for tumor weight and lung-body-weight ratio in the subcutaneous or intravenous model by Mann-Whitney (E: U=32, p=0.4997; H: U=3, p=0.3429), for tumor volume in the subcutaneous model by multiple t-tests and for metastasis count and area per slide by unpaired t-test (I: df=5, t=0.1362, p=0.4152; J: df=5, t=1.644, p=0.1610).

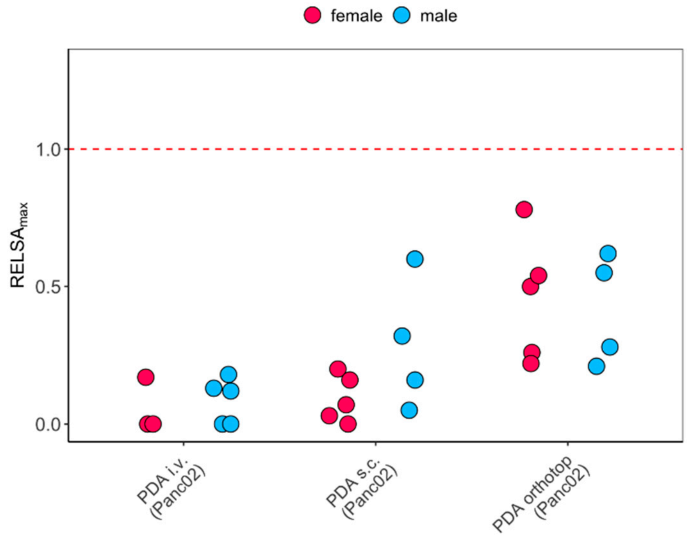


**Figure S2.** Comparison of $RELSA_{max}$ values for sex related differences. The $RELSA_{max}$ estimates of either female (red) or male (blue) mice were graphed separately for the intravenous (i.v.), subcutaneous (s.c.) and orthotopic (orthotop) pancreatic cancer model, induced with Panc02 cells. No significant differences of the individual $RELSA_{max}$ estimates between male and female mice were observed within each model by ANOVA ($F(1)= [0.0788], p=0.3852$).

**Table S1.** Clinical score sheet

| Observations | Score |
|---|---|
| **I Body weight** | |
| I-a decreased > 10% (compared to initial weight) | 3 |
| I-b decreased > 15% (compared to initial weight) | 4 |

| Observations | Score |
|---|---|
| **II General condition** | |
| appearance | 1 (A) |
| II-a tooth displacement, too long teeth | 2 |
| II-b fur dull, ruffled or untended | 2 |
| II-c eyes unclear or squinted | 3 |
| II-d untended orifices of the body | 3 |
| II-e abnormal posture | 3 |
| II-f dehydration | |
| impairments/abnormalities | 3 |
| II-g short spasms or temporary paralysis symptoms | 4 |
| II-h persistent (>30’) cramping or paralysis | |
| II-i abnormal respiratory sounds or breathing, | 4 |
| animal feels cold | |
| II-j state of cachexia (body condition score: BC 2) | 4 |

| Observations | Score |
|---|---|
| **III Spontaneous behavior** | |
| III-a animal is passive or overactive | 2 |
| III-b pronounced apathy, hyperkinesia, isolation | 4 |
| III-c squeaking due to pain | 4 |
| III-d self-mutilation | 4 |

| Observations | Score |
|---|---|
| **IV Flight behavior after contact** | |
| IV-a animal is moderately passive or overactive | 2 |
| IV-b distinct apathy or hyperkinesia | 4 |

| Observations | Score |
|---|---|
| **V Process-specific criteria** | |
| V-a wound healing disorder | 2 |
| V-b opening of the sutures by biting | 1 (B) |
| V-c local inflammation | 2 |
| V-d ascites | 4 |
| V-e rectal bleeding/anal prolapse | 4 |
| V-f tumor size $\geqslant$ 2000 $m^3$ | 4 |
| V-g tumor-related impairment of motion | 4 |
| V-h tumor ulceration | 4 |
| V-i bloated belly and bent body posture | 4 |

Score points given per line as soon as one criteria applies. Even with several positive results per line,there is no addition of the points per line.

**Procedures**

| Single score | Total score | Distress level | Measures |
|---|---|---|---|
| A | | mild | Shorten teeth. |
| B | | moderate | Inform the person in charge of the experiment. If necessary, anesthetize the animal and close the wound. Document it. |
| 1 | | mild | Inform the person in charge of the experiment. A sufficient frequency of observation is necessary, consider treatment options and document it. |
| 2-3 | | moderate | Inform the person in charge of the experiment. Daily observation of the animal is necessary, consider treatment options and document it. |
| 4 | | severe | In agreement with the person in charge euthanasia (preferably painless after anesthesia) has to be performed. Document it. |
| | 3-4 | mild | Inform the person in charge of the experiment. Daily observation of the animal is necessary, consider treatment options and document it. |
| | 5-10 | moderate | Inform the person in charge of the experiment. Euthanasia or treating the animal plus daily observation of the animal is necessary. Document it. |
| | > 11 | severe | In agreement with the person in charge euthanasia (preferably painless after anesthesia) has to be performed. Document it. |

**Table S2.** Severity assessment parameters for cancer cell injection in the orthotopic model divided by sex.

| | Sex | N | | pre vs. days after tumor cell injection | | | | |
|---|---|---|---|---|---|---|---|---|
| | | | | pre | 0 | 1 | 2 | 3 |
| **body weight** | ♀ | 5 | Mean | -0.472 | -4.222 | -2.436 | 0.016 | -0.644 |
| | | | ±SD | 2.062 | 3.659 | 1.693 | 1.359 | 1.546 |
| | ♂ | 4 | Mean | -0.363 | -1.123 | -3.200 | -3.128 | -2.685 |
| | | | ±SD | 2.174 | 1.670 | 0.855 | 5.431 | 4.762 |
| **distress score** | ♀ | 5 | Mean | 0.000 | 0.400 | 1.000 | 0.000 | 0.000 |
| | | | ±SD | 0.000 | 0.894 | 2.236 | 0.000 | 0.000 |
| | ♂ | 4 | Mean | 0.000 | 0.500 | 0.000 | 0.750 | 0.000 |
| | | | ±SD | 0.000 | 1.000 | 0.000 | 1.500 | 0.000 |
| **perianal temperature** | ♀ | 5 | Mean | 31.120 | 31.560 | 31.100* | 30.740 | 30.840 |
| | | | ±SD | 0.716 | 0.643 | 0.543 | 0.623 | 0.796 |
| | ♂ | 4 | Mean | 30.325 | 30.200 | 29,900* | 30,225 | 29,975 |
| | | | ±SD | 0.222 | 0.812 | 0.141 | 0.275 | 0.411 |
| **burrowing (2 hours)** | ♀ | 3 | Mean | 135.333 | 66.667 | 81.000 | 133.667 | 118.667 |
| | | | ±SD | 26.577 | 34.122 | 47.697 | 32.083 | 65.684 |
| | ♂ | 3 | Mean | 181.00 | 36.000 | 168.333 | 126.000 | 166.000 |
| | | | ±SD | 22.605 | 51.507 | 42.525 | 37.987 | 24.556 |
| **burrowing (17 hours)** | ♀ | 4 | Mean | 191.000 | 168.000 | 196.250 | 199.250 | 198.750 |
| | | | ±SD | 10.424 | 56.798 | 6.238 | 0.957 | 1.893 |
| | ♂ | 3 | Mean | 192.000 | 177.333 | 195.000 | 193.667 | 200.000 |
| | | | ±SD | 13.856 | 21.595 | 8.660 | 10.970 | 0.000 |
| **nesting** | ♀ | 2 | Mean | 4.000 | 2.500 | 4.000 | 5.000# | 5.000 |
| | | | ±SD | 0.000 | 0.707 | 1.414 | 0.000 | 1.414 |
| | ♂ | 3 | Mean | 5.000 | 4.000 | 5.667 | 4.000 | 4.333 |
| | | | ±SD | 1.000 | 2.646 | 0.577 | 2.000 | 2.887 |
| **mouse grimace scale** | ♀ | 5 | Mean | 0.000 | 0.244 | -0.080 | -0.080 | -0.027 |
| | | | ±SD | 0.000 | 0.356 | 0.177 | 0.138 | 0.135 |
| | ♂ | 4 | Mean | 0.000 | 0.272 | 0.061 | 0.011 | 0.067 |
| | | | ±SD | 0.000 | 0.175 | 0.192 | 0.056 | 0.077 |
| **fecal corticosterone metabolites** | ♀ | 5 | Mean | 71.240 | 130.920 | 120.180# | 91.440 | 84.920 |
| | | | ±SD | 20.972 | 56.008 | 40.725 | 26.442 | 14.801 |
| | ♂ | 4 | Mean | 72.200 | 153.150 | 141.025 | 135.650 | 86.125 |
| | | | ±SD | 40.260 | 118.409 | 58.412 | 94.551 | 49.901 |

Analysis by two-way ANOVA: # p≤0.05 comparison to pre value, * p≤0.05 pairwise comparison of both sex at a certain time point.

**Table S3.** Severity assessment parameters for cancer cell injection in the intravenous model divided by sex.

| | Sex | N | | pre vs. days after tumor cell injection | | | | |
|---|---|---|---|---|---|---|---|---|
| | | | | **pre** | **0** | **1** | **2** | **3** |
| **body weight** | ♀ | 3 | Mean | 2.477 | 2.610 | 1.490 | 1.983 | 3.707 |
| | | | ±SD | 3.089 | 4.570 | 6.384 | 4.504 | 2.967 |
| | ♂ | 5 | Mean | 2.014 | 2.308 | -0.840 | 1.522 | 1.348 |
| | | | ±SD | 0.818 | 1.371 | 5.001 | 2.229 | 1.761 |
| **distress score** | ♀ | 3 | Mean | 0.000 | 0.000 | 0.000 | 0.000 | 0.000 |
| | | | ±SD | 0.000 | 0.000 | 0.000 | 0.000 | 0.000 |
| | ♂ | 5 | Mean | 0.000 | 0.000 | 0.000 | 0.000 | 0.000 |
| | | | ±SD | 0.000 | 0.000 | 0.000 | 0.000 | 0.000 |
| **perianal temperature** | ♀ | 3 | Mean | 30.667 | 30.967 | 30.167 | 30.200 | 30.500 |
| | | | ±SD | 0.289 | 0.945 | 0.416 | 0.557 | 0.100 |
| | ♂ | 5 | Mean | 29.940 | 29.640 | 29.600 | 29.640 | 29.440 |
| | | | ±SD | 0.623 | 0.631 | 0.361 | 0.680 | 0.619 |
| **burrowing (2 hours)** | ♀ | 2 | Mean | 104.500 | 47.500 | 68.000 | 28.000# | 18.000* |
| | | | ±SD | 3.536 | 43.134 | 28.284 | 5.657 | 15.556 |
| | ♂ | 4 | Mean | 176.500 | 180.000 | 166.500 | 128.250# | 141.500* |
| | | | ±SD | 31.395 | 34.871 | 44.486 | 36.818 | 32.970 |
| **burrowing (17 hours)** | ♀ | 3 | Mean | 200.000 | 181.000 | 189.667 | 181.333 | 183.000 |
| | | | ±SD | 0.000 | 21.284 | 17.898 | 32.332 | 17.000 |
| | ♂ | 5 | Mean | 195.600 | 199.800 | 174.400 | 199.200 | 171.200 |
| | | | ±SD | 8.735 | 0.447 | 35.402 | 1.789 | 19.665 |
| **nesting** | ♀ | 2 | Mean | 4.500 | 3.000 | 1.500# | 2.000 | 3.500# |
| | | | ±SD | 0.707 | 2.828 | 0.707 | 1.414 | 0.707 |
| | ♂ | 5 | Mean | 5.200 | 5.200 | 4.600 | 4.400 | 4.800 |
| | | | ±SD | 0.447 | 0.837 | 0.548 | 1.517 | 0.837 |
| **mouse grimace scale** | ♀ | 3 | Mean | 0.000 | 0.081 | -0.007 | 0.119 | 0.126 |
| | | | ±SD | 0.000 | 0.148 | 0.078 | 0.173 | 0.103 |
| | ♂ | 5 | Mean | 0.000 | 0.111 | 0.053 | 0.044 | 0.040 |
| | | | ±SD | 0.000 | 0.167 | 0.116 | 0.121 | 0.053 |
| **fecal corticosterone metabolites** | ♀ | 3 | Mean | 192.300 | 380.033 | 270.967 | 267.233 | 277.500 |
| | | | ±SD | 59.601 | 255.796 | 142.026 | 102.245 | 159.854 |
| | ♂ | 5 | Mean | 99.480 | 121.280 | 110.320 | 124.860 | 110.060 |
| | | | ±SD | 34.200 | 39.422 | 35.946 | 16.477 | 11.991 |

Analysis by two-way ANOVA: # p≤0.05 comparison to pre value, * p≤0.05 pairwise comparison of both sex at a certain time point.

**Table S4.** Severity assessment parameters for cancer cell injection in the subcutaneous model divided by sex.

| | Sex | N | | pre vs. days after tumor cell injection | | | | |
|---|---|---|---|---|---|---|---|---|
| | | | | **pre** | **0** | **1** | **2** | **3** |
| **body weight** | ♀ | 5 | Mean | 0.570 | 1.750 | 1.712 | 1.992 | 2.356 |
| | | | ±SD | 3.265 | 3.127 | 3.605 | 4.400 | 3.909 |
| | ♂ | 4 | Mean | -0.993 | -0.575 | -0.038 | -0.105 | -1.810 |
| | | | ±SD | 1.955 | 1.744 | 3.284 | 4.221 | 6.818 |
| **distress score** | ♀ | 5 | Mean | 0.000 | 0.000 | 0.000 | 0.000 | 0.000 |
| | | | ±SD | 0.000 | 0.000 | 0.000 | 0.000 | 0.000 |
| | ♂ | 4 | Mean | 0.000 | -0.500 | 0.000 | 0.000 | 0.000 |
| | | | ±SD | 0.000 | 1.000 | 0.000 | 0.000 | 0.000 |
| **perianal temperature** | ♀ | 5 | Mean | 30.680 | 28.640#* | 30.860 | 30.940 | 30.760 |
| | | | ±SD | 0.130 | 0.439 | 0.688 | 0.850 | 0.518 |
| | ♂ | 4 | Mean | 30.775 | 30.625* | 30.200# | 30.325 | 29.850 |
| | | | ±SD | 0.359 | 0.263 | 0.216 | 0.126 | 0.173 |
| **burrowing (2 hours)** | ♀ | 4 | Mean | 145.750 | 166.750 | 142.500 | 139.750* | 77.500 |
| | | | ±SD | 39.424 | 30.369 | 66.521 | 41.492 | 30.881 |
| | ♂ | 3 | Mean | 120.667 | 103.667 | 140.000 | 190.333* | 200.000 |
| | | | ±SD | 21.548 | 96.914 | 70.064 | 16.743 | 0.000 |
| **burrowing (17 hours)** | ♀ | 4 | Mean | 200.000 | 195.000 | 196.000 | 197.750 | 195.500 |
| | | | ±SD | 0.000 | 10.000 | 8.000 | 4.500 | 9.000 |
| | ♂ | 4 | Mean | 198.500 | 190.000 | 185.750 | 173.000 | 166.000 |
| | | | ±SD | 2.380 | 15.144 | 22.897 | 54.000 | 68.000 |
| **nesting** | ♀ | 3 | Mean | 5.000 | 5.333 | 4.667 | 5.667 | 5.333 |
| | | | ±SD | 1.000 | 1.155 | 2.309 | 0.577 | 0.577 |
| | ♂ | 4 | Mean | 5.000 | 4.250 | 4.750 | 4.750 | 4.750 |
| | | | ±SD | 0.816 | 1.258 | 0.957 | 0.957 | 1.500 |
| **mouse grimace scale** | ♀ | 5 | Mean | 0.000 | 0.027 | 0.018 | 0.058 | 0.018 |
| | | | ±SD | 0.000 | 0.099 | 0.101 | 0.141 | 0.119 |
| | ♂ | 4 | Mean | 0.000 | 0.189 | 0.078 | 0.039 | 0.100 |
| | | | ±SD | 0.000 | 0.115 | 0.086 | 0.066 | 0.082 |
| **fecal corticosterone metabolites** | ♀ | 5 | Mean | 102.160* | 139.380* | 144.360 | 145.940 | 162.700* |
| | | | ±SD | 19.267 | 21.376 | 41.707 | 48.986 | 49.860 |
| | ♂ | 4 | Mean | 54.700* | 60.150* | 93.825 | 91.350 | 58.500* |
| | | | ±SD | 11.840 | 17.832 | 33.632 | 57.928 | 36.151 |

Analysis by two-way ANOVA: # p≤0.05 comparison to pre value, * p≤0.05 pairwise comparison of both sex at a certain time point.

**Table S5.** ANOVA tables for analysis of severity assessment parameters on perioperative days (Figure 2).

| | | SS | df | MS | F ($df_n$, $df_d$) | p value |
|---|---|---|---|---|---|---|
| **body weight (ANOVA)** | time x PDA model | 75.26 | 8 | 9.407 | F (8, 92) = 2.200 | 0.0343 |
| | time | 35.17 | 4 | 8.792 | F (2.652, 61.00) = 2.056 | 0.1224 |
| | PDA model | 300 | 2 | 150 | F (2, 23) = 3.935 | 0.0339 |
| | mouse | 876.8 | 23 | 38.12 | F (23, 92) = 8.917 | <0.0001 |
| | Residual | 393.3 | 92 | 4.275 | | |
| **perianal temperature (ANOVA)** | time x PDA model | 7.923 | 8 | 0.9904 | F (8, 92) = 2.678 | 0.0108 |
| | time | 2.06 | 4 | 0.5151 | F (2.007, 46.15) = 1.393 | 0.2587 |
| | PDA model | 9.924 | 2 | 4.962 | F (2, 23) = 3.817 | 0.0370 |
| | mouse | 29.9 | 23 | 1.3 | F (23, 92) = 3.515 | <0.0001 |
| | Residual | 34.03 | 92 | 0.3698 | | |
| **nesting (ANOVA)** | time x PDA model | 11.02 | 8 | 1.378 | F (8, 64) = 1.981 | 0.0631 |
| | time | 6.461 | 4 | 1.615 | F (3.349, 53.58) = 2.322 | 0.0790 |
| | PDA model | 7.651 | 2 | 3.826 | F (2, 16) = 0.7439 | 0.4910 |
| | mouse | 82.29 | 16 | 5.143 | F (16, 64) = 7.394 | <0.0001 |
| | Residual | 44.51 | 64 | 0.6955 | | |
| **burrowing (2 hours, ANOVA)** | time x PDA model | 45427 | 8 | 5678 | F (8, 64) = 3.058 | 0.0056 |
| | time | 15586 | 4 | 3897 | F (2.816, 45.05) = 2.099 | 0.1173 |
| | PDA model | 8189 | 2 | 4095 | F (2, 16) = 0.5029 | 0.6140 |
| | mouse | 130265 | 16 | 8142 | F (16, 64) = 4.385 | <0.0001 |
| | Residual | 118834 | 64 | 1857 | | |
| **burrowing (17 hours, ANOVA)** | time x PDA model | 5772 | 8 | 721.5 | F (8, 80) = 1.736 | 0.1027 |
| | time | 1654 | 4 | 413.5 | F (2.523, 50.46) = 0.9947 | 0.3923 |
| | PDA model | 249.2 | 2 | 124.6 | F (2, 20) = 0.1196 | 0.8879 |
| | mouse | 20831 | 20 | 1042 | F (20, 80) = 2.506 | 0.0020 |
| | Residual | 33252 | 80 | 415.7 | | |
| **mouse grimace scale (ANOVA)** | time x PDA model | 0.2321 | 8 | 0.02902 | F (8, 92) = 2.342 | 0.0245 |
| | time | 0.3667 | 4 | 0.09168 | F (2.851, 65.58) = 7.400 | 0.0003 |
| | PDA model | 0.002146 | 2 | 0.001073 | F (2, 23) = 0.03283 | 0.9677 |
| | mouse | 0.7518 | 23 | 0.03269 | F (23, 92) = 2.638 | 0.0006 |
| | Residual | 1.14 | 92 | 0.01239 | | |
| **fecal corticosterone metabolites (ANOVA)** | time x PDA model | 19499 | 8 | 2437 | F (8, 92) = 1.149 | 0.3388 |
| | time | 50849 | 4 | 12712 | F (2.521, 57.99) = 5.992 | 0.0022 |
| | PDA model | 122491 | 2 | 61245 | F (2, 23) = 2.429 | 0.1104 |
| | mouse | 579972 | 23 | 25216 | F (23, 92) = 11.89 | <0.0001 |
| | Residual | 195171 | 92 | 2121 | | |

**Table S6.** ANOVA tables for analysis of severity assessment parameters for tumor progression (Figure 3).

| | | SS | df | MS | F ($df_n$, $df_d$) | p value |
|---|---|---|---|---|---|---|
| **body weight (ANOVA)** | time x PDA model | 39.45 | 6 | 6.575 | F (6, 63) = 1.791 | 0.1152 |
| | time | 109.3 | 3 | 36.44 | F (2.785, 58.48) = 9.929 | <0.0001 |
| | PDA model | 142 | 2 | 70.99 | F (2, 21) = 2.791 | 0.0842 |
| | mouse | 534.1 | 21 | 25.43 | F (21, 63) = 6.930 | <0.0001 |
| | Residual | 231.2 | 63 | 3.67 | | |
| **perianal temperature (ANOVA)** | time x PDA model | 2.41 | 6 | 0.4016 | F (6, 63) = 1.452 | 0.2094 |
| | time | 3.752 | 3 | 1.251 | F (2.552, 53.59) = 4.520 | 0.0096 |
| | PDA model | 3.652 | 2 | 1.826 | F (2, 21) = 2.333 | 0.1216 |
| | mouse | 16.43 | 21 | 0.7826 | F (21, 63) = 2.829 | 0.0008 |
| | Residual | 17.43 | 63 | 0.2767 | | |
| **nesting (ANOVA)** | time x PDA model | 5.867 | 6 | 0.9778 | F (6, 45) = 0.9208 | 0.4891 |
| | time | 9.824 | 3 | 3.275 | F (2.132, 31.98) = 3.084 | 0.0566 |
| | PDA model | 0.9107 | 2 | 0.4554 | F (2, 15) = 0.1107 | 0.8960 |
| | mouse | 61.71 | 15 | 4.114 | F (15, 45) = 3.874 | 0.0002 |
| | Residual | 47.79 | 45 | 1.062 | | |
| **burrowing (2 hours, ANOVA)** | time x PDA model | 8918 | 6 | 1486 | F (6, 48) = 1.953 | 0.0913 |
| | time | 14013 | 3 | 4671 | F (2.693, 43.08) = 6.136 | 0.0020 |
| | PDA model | 7166 | 2 | 3583 | F (2, 16) = 2.126 | 0.1518 |
| | mouse | 26965 | 16 | 1685 | F (16, 48) = 2.214 | 0.0174 |
| | Residual | 36538 | 48 | 761.2 | | |
| **burrowing (17 hours, ANOVA)** | time x PDA model | 427.3 | 6 | 71.21 | F (6, 57) = 0.8292 | 0.5523 |
| | time | 96.76 | 3 | 32.25 | F (1.559, 29.63) = 0.3755 | 0.6382 |
| | PDA model | 831.1 | 2 | 415.5 | F (2, 19) = 3.772 | 0.0417 |
| | mouse | 2093 | 19 | 110.2 | F (19, 57) = 1.283 | 0.2310 |
| | Residual | 4895 | 57 | 85.88 | | |
| **fecal corticosterone metabolites (ANOVA)** | time x PDA model | 8328 | 6 | 1388 | F (6, 63) = 0.8029 | 0.5714 |
| | time | 21153 | 3 | 7051 | F (2.171, 45.59) = 4.079 | 0.0208 |
| | PDA model | 5738 | 2 | 2869 | F (2, 21) = 0.2337 | 0.7937 |
| | mouse | 257847 | 21 | 12278 | F (21, 63) = 7.103 | <0.0001 |
| | Residual | 108902 | 63 | 1729 | | |

**Table S7.** ANOVA tables for analysis of severity assessment parameters for humane endpoint phase (Figure 4).

| | | SS | df | MS | F ($df_n$, $df_d$) | p value |
|---|---|---|---|---|---|---|
| **body weight (ANOVA)** | time x PDA model | 254.8 | 10 | 25.48 | F (10, 100) = 1.901 | 0.0536 |
| | time | 1206 | 5 | 241.3 | F (3.318, 66.37) = 18.00 | <0.0001 |
| | PDA model | 780.8 | 2 | 390.4 | F (2, 20) = 4.718 | 0.0210 |
| | mouse | 1655 | 20 | 82.75 | F (20, 100) = 6.174 | <0.0001 |
| | Residual | 1340 | 100 | 13.4 | | |
| **distress score (ANOVA)** | time x PDA model | 90.17 | 10 | 9.017 | F (10, 100) = 4.582 | <0.0001 |
| | time | 934.4 | 5 | 186.9 | F (2.744, 54.88) = 94.97 | <0.0001 |
| | PDA model | 37.61 | 2 | 18.81 | F (2, 20) = 5.220 | 0.0150 |
| | mouse | 72.05 | 20 | 3.603 | F (20, 100) = 1.831 | 0.0269 |
| | Residual | 196.8 | 100 | 1.968 | | |
| **corticosterone in plasma (ANOVA)** | time x PDA model | 101398 | 2 | 50699 | F (2, 18) = 7.349 | 0.0046 |
| | time | 201190 | 1 | 201190 | F (1, 18) = 29.16 | <0.0001 |
| | PDA model | 115814 | 2 | 57907 | F (2, 18) = 6.294 | 0.0085 |
| | mouse | 165605 | 18 | 9200 | F (18, 18) = 1.334 | 0.2738 |
| | Residual | 124170 | 18 | 6898 | | |
